# Control of Transmembrane Protein Diffusion within the Postsynaptic Density Assessed by Simultaneous Single-Molecule Tracking and Localization Microscopy

**DOI:** 10.3389/fnsyn.2016.00019

**Published:** 2016-07-22

**Authors:** Tuo P. Li, Thomas A. Blanpied

**Affiliations:** Department of Physiology and Program in Neuroscience, University of Maryland School of MedicineBaltimore, MD, USA

**Keywords:** uPAINT, PALM, synapse, nanodomain, glutamate receptor, macromolecular crowding

## Abstract

Postsynaptic transmembrane proteins are critical elements of synapses, mediating trans-cellular contact, sensitivity to neurotransmitters and other signaling molecules, and flux of Ca and other ions. Positioning and mobility of each member of this large class of proteins is critical to their individual function at the synapse. One critical example is that the position of glutamate receptors within the postsynaptic density (PSD) strongly modulates their function by aligning or misaligning them with sites of presynaptic vesicle fusion. In addition, the regulated ability of receptors to move in or out of the synapse is critical for activity-dependent plasticity. However, factors that control receptor mobility within the boundaries of the synapse are not well understood. Notably, PSD scaffold molecules accumulate in domains much smaller than the synapse. Within these nanodomains, the density of proteins is considerably higher than that of the synapse as a whole, so high that steric hindrance is expected to reduce receptor mobility substantially. However, while numerical modeling has demonstrated several features of how the varying protein density across the face of a single PSD may modulate receptor motion, there is little experimental information about the extent of this influence. To address this critical aspect of synaptic organizational dynamics, we performed single-molecule tracking of transmembrane proteins using universal point accumulation-for-imaging-in-nanoscale-topography (uPAINT) over PSDs whose internal structure was simultaneously resolved using photoactivated localization microscopy (PALM). The results provide important experimental confirmation that PSD scaffold protein density strongly influences the mobility of transmembrane proteins. A protein with a cytosolic domain that does not bind PSD-95 was still slowed in regions of high PSD-95 density, suggesting that crowding by scaffold molecules and perhaps other proteins is sufficient to stabilize receptors even in the absence of binding. Because numerous proteins thought to be involved in establishing PSD structure are linked to disorders including autism and depression, this motivates further exploration of how PSD nanostructure is created. The combined application PALM and uPAINT should be invaluable for distinguishing the interactions of mobile proteins with their nano-environment both in synapses and other cellular compartments.

## Introduction

Transmembrane proteins such as receptors diffuse on the cell surface to reach their sites of action. In doing so, they must make their way through complex environments typified by varying densities of obstacles and potential binding partners. The average behavior of proteins moving through such environments has been well characterized (Frick et al., 2007). However, on small spatial scales or within small compartments, the local organization of potential interactors will dominate the influence on receptor motion paths (Kusumi et al., 2014). For instance, locally high concentrations of steric obstacles create a phenomenon called macromolecular crowding (Ryan et al., 1988) that can slow mobility and result in anomalous diffusion (Saxton, 1994; Santamaria et al., 2010). Thus, high-resolution information about the distribution of even non-binding obstacles is necessary to understand motion trajectories of transmembrane proteins on small scales.

Perhaps the most complex compartment of the plasma membrane in neurons is the postsynaptic density (PSD). The PSD of glutamatergic synapses concentrates numerous receptor types aligned to the presynaptic active zone. Despite the small size of the average PSD (~0.08 μm^2^ × 50 nm; Harris and Weinberg, 2012), roughly 500 species of proteins can be found in this compartment (Husi et al., 2000; Sheng and Hoogenraad, 2007). Because of the high local density of transmembrane proteins, receptor-binding proteins such as PSD-95, and juxtamembrane cytosolic molecules, protein motion within the membrane at the synapse is likely extremely obstructed (Santamaria et al., 2010). This complicated environment is critical to understand, because protein organization in the PSD directly regulates synaptic transmission in many ways. The number of glutamate receptors present in the PSD sets an upper limit on the strength of the synapse (Huganir and Nicoll, 2013), and receptors exchange continuously by diffusion between the PSD and the perisynaptic plasma membrane (Opazo and Choquet, 2011; Choquet and Triller, 2013). Further, alterations to the PSD are a critical component of activity-driven plasticity mechanisms regulating receptor number (Inoue and Okabe, 2003; Bosch et al., 2014). Thus, understanding mechanisms within the PSD that control motion of glutamate receptors is critical for determining how receptor number is modulated during plasticity.

Even beyond the clear importance of the number of receptors, however, their distribution within the synapse in the plane of the membrane is a vital regulator of synaptic strength (MacGillavry et al., 2011). This is because when glutamatergic vesicles fuse with the presynaptic plasma membrane, the result is a highly concentrated but narrow spike of released neurotransmitter. The rapid dissipation of this spike by glutamate diffusion means that receptors laterally displaced from the site of fusion even by less than 100 nm often fail to activate (Xie et al., 1997; Raghavachari and Lisman, 2004; Santucci and Raghavachari, 2008; Freche et al., 2011). Amplifying this effect, receptors in the PSD are concentrated in ~80 nm subdomains (MacGillavry et al., 2013; Nair et al., 2013) where the principle receptor-binding scaffold PSD-95 is also concentrated (Fukata et al., 2013; MacGillavry et al., 2013). In previous work, we modeled diffusion within PSDs where the heterogeneous distribution of PSD-95 was measured, and found that the clustered nature of this scaffold could strongly limit the ability of transmembrane proteins to enter (or escape) the crowded regions of the PSD (Li et al., 2016). Thus, nanoscale regional variation in protein composition within a single PSD may have strong impact on synaptic transmission by controlling the subsynaptic distribution of receptors.

A major impediment to progress on this issue is the technical challenge of simultaneously measuring the nanoscale distribution of the protein environment while simultaneously measuring protein motion through it. To address this, we developed a combined single-molecule imaging approach that uses single-particle tracking photoactivated localization microscopy (sptPALM; Manley et al., 2008) to map the positions of PSD-95 molecules within the synapse (MacGillavry et al., 2013), while simultaneously tracking the motion of proteins in the plasma membrane by universal point-accumulation-for-imaging-in-nanoscale-topography (uPAINT; Giannone et al., 2010). Using this strategy, we could directly investigate the influence of both obstacle density and protein binding on motion through the PSD. The results provide direct experimental confirmation that macromolecular crowding within the PSD can strongly limit the motion of even small transmembrane proteins, likely helping to establish the distribution and dynamic exchange characteristics of glutamate receptors and other molecules.

## Materials and Methods

### Neuron Culture and Transfection

Dissociated hippocampal neuron cultures were prepared from E18 rat embryos as described previously (Frost et al., 2010). All procedures conformed to the guidelines established by Animal Welfare Act, Public Health Service, and the United States Department of Agriculture, and were approved by the Institutional Animal Care and Use Committee at the University of Maryland, Baltimore. Prior to plating the cells on coverslips, the coverslips were first cleaned as reported previously (MacGillavry et al., 2013), subsequently coated with lateral-drift tracking, yellow-green fluorescent 100 nm beads (F8803; Thermo Fischer Scientific) diluted 1:25,000 in 100% ethanol (dried within 25 min in the hood), and then coated overnight with poly-L-lysine (Sigma). Cells were transfected at DIV10–13 using Lipofectamine 2000 (Thermo Fischer Scientific) and imaged 72–96 h later (unless stated otherwise). Individual coverslips were transfected with 0.5–0.75 μg of cDNA for each expression construct.

### Expression Constructs

Plasmid cDNAs were obtained as follows (with original sources): the binding and nonbinding probes, Super ecliptic phluorin (SEP)-transmembrane (TM)-Bind and SEP-TM-Nonbind (Li et al., 2016), the PSD-95-mEos2 replacement plasmid shrPSD-95-mEos2 (MacGillavry et al., 2013). We have previously measured that this construct produces a mild 1.4× overexpression. Interpretations of the results from the PALM-PAINT assay require control experiments to ensure that the expressed protein is organized similarly to its endogenous counterpart. To this end, we have also compared the PSD-95 nanostructure of untransfected cells and those expressing the replacement construct PSD-95-mEos2 (MacGillavry et al., 2013; Tang et al., in press). These were not different in terms of PSD area, number of subsynaptic nanoclusters, and the size of those nanoclusters. Thus, we believe the mEos2 tag does not affect PSD nanostructure, at least in the absence of severe PSD-95 overexpression. Furthermore, mEos2 is fused at the c-terminal end of the PSD-95, not altering the positions of the various protein-protein interaction motifs. Thus, we believe tagging itself does not affect the interaction of PSD-95 with other proteins.

### Two-Color Single-Molecule Imaging

PALM-PAINT, a combination of PALM (Betzig et al., 2006; Hess et al., 2006) and uPAINT (Giannone et al., 2010), was performed through a Photometrics DV2 on an Olympus IX81 ZDC2 inverted microscope that was described by MacGillavry et al. (2013). Cells expressing the indicated constructs were imaged in a previously described extracellular buffer (Li et al., 2016). SEP-containing probes were labeled with ATTO647N-conjugated anti-green fluorescent protein (GFP) nanobodies (GFPBooster-647N, Chromotek), bath applied to a final concentration of 0.5–2 nM once the first stretch of synapses was identified for each coverslip. Cells remained at 25°C for no more than 30 min per imaging session.

We imaged the red and far-red bands by interleaving excitations of 561 and 640 nm. Imaging was conducted at 29 Hz (14.5 Hz per color), with 10-ms duration excitation per frame for 5000–20,000 frames per color. The two emissions were overlaid based on calibration images of TetraSpeck beads (100 nm; Thermo Fischer Scientific) deposited on an acellular coverslip as described by MacGillavry et al. (2013). Yellow-green beads (100 nm) ethanol-diluted and dried onto the coverslips prior to plating the cells (F8803; Thermo Fischer Scientific) were excited and captured once every 1000 frames to monitor lateral drift. To correct lateral drift, we localized fiducials *post hoc* from images of the yellow-green beads. We screened for spurious localizations by the duration of fluorescence and mobility. Namely, the bead ought to be present on the first frame, persist for as long as each imaging session, and displace <100 nm (1 pixel) per 1000 frames. Such a filtering process provided a list of localizations that correspond to fiducials. From this list we calculated the sample lateral drift as the weighted average of the displacements of all fiduciary localizations between each set of 1000 frames. For weights, we used the inverse of the estimated localization uncertainty (Thompson et al., 2002) of each fiducial. The single linear correction in drift we applied to each subset of 1000 frames was the average correction obtained from the estimates of 2–10 fiducials in the field of view.

### Single-Molecule Localization, Tracking Analysis, and PSD Nanostructure Analysis

All data analysis was performed offline using custom routines in MATLAB (The MathWorks). The algorithms for determining molecule location and criteria for filtering molecules to be considered for further analysis were applied as previously described (MacGillavry et al., 2013). In addition to filtering by localization precision, elliptical form, and brightness, we also utilized a Voronoi-based segmentation program SR-Tesseler (Levet et al., 2015) to filter out spurious localizations outside of putative neuronal border. Criteria for defining a track were described by Li et al. (2016). Instantaneous effective diffusion coefficients (D_eff_) at individual track time points were calculated for tracks that persisted at least eight frames (the duration in which the mean mean squared displacement (MSD) was linear; a more detailed description can be found in Lu et al., 2014). For comparing diffusion inside and outside of PSDs, tracks that entered or exited the PSD were divided into two portions, a synaptic and an extrasynaptic subtrack, the D_eff_ of which were calculated by averaging the instantaneous D_eff_ for the tracked localizations therein. For the rare tracks that entered and exited PSDs multiple times, synaptic D_eff_ was determined by averaging the instantaneous D_eff_ of all the tracked locations inside the PSD border; vice versa to calculate the extrasynaptic D_eff_. To calculate the D_eff_ of subtracks in other cases (e.g., within a particular range of PSD-95 regional density, or below the detection limit of D_eff_), we calculated the average of instantaneous D_eff_ of localizations meeting the criteria. The lower detection limit of D_eff_ was determined conservatively by calculating the D_eff_ of a theoretical immobile particle displaced as much as the average trajectory error ~30 nm (Savin and Doyle, 2005; Lu et al., 2014) per time frame, which amounted to 0.003 μm^2^/s; this value is indicated by a gray vertical dotted or dotted-dashed line in cumulative frequency graphs of D_eff_.

The PALM PSD border was determined by taking the convex hull of the PSD-95 molecular positions. To determine the Gaussian-blurred border of each PSD, we first constructed a 2-dimensional molecular density map of 25 × 25 nm subpixels from PSD-95 molecules for each PSD. We then convolved it with a constant-amplitude Gaussian image profile (*σ* = 125 nm, image width = 900 nm) that is similar to an ideal microscope point-spread function with a full-width at half max (FWHM) of ~250 nm. To determine the FWHM border of the blurred PSD, we thresholded the convolved image at half-maximum intensity. To determine the 95%-border, we thresholded it at 5% of the maximum intensity.

### Determination of Regional PSD-95 Density

To quantify the regional density of PSD-95, we measured the number of molecules surrounding each position of a tracked probe molecule. PSD-95 localizations appearing in consecutive frames separated by no more than 200 nm were considered one molecule, and its position was taken from the first frame it appeared. We counted the number of PSD-95 molecules within a 30 nm radius (the average trajectory error) of each position in the track of a probe molecule. Because we wished primarily to relate D_eff_ to regional density, we used the same tracked locations to calculate each measure. That is, for the regional density of PSD-95 surrounding the probe at each position, we calculated the forward running average of regional densities for eight frames, the same number of frames used to calculate D_eff_. The absolute density of molecules in all calculations was adjusted by the average expected number of blinks (one) of mEos2 in our experimental conditions (Annibale et al., 2011; MacGillavry et al., 2013). The final density when averaged across synapses is likely somewhat higher than endogenous PSD-95 densities due to the mild (~1.4-fold) overexpression obtained with our shRNA knockdown/replacement approach. However, we did not attempt to measure the potentially quite variable ratio of endogenous and tagged PSD-95 at each synapse analyzed, and did not take this into account for calculations. Data of immunostaining done in cells not intended for PALM-PAINT showed that endogenous PSD-95 molecules resistant to the knockdown did not form dense clusters outside those formed by the expressed PSD-95 molecules, indicating that though the endogenous PSD-95 might be present in the synapse, they likely raise the absolute number of PSD-95 molecules without changing the spatial variation in regional density.

### Statistics

Where means are presented, the accompanying errors are the standard error of the mean; additionally, these data were normally distributed according to the Shapiro-Wilk normality test. Where box-and-whisker plots are presented, the middle bar represents the median, the upper and lower limit of the boxes denote the interquartile range, and the whiskers extend to 5% and 95% of the distribution; additionally, these data were not normally distributed according to the Shapiro-Wilk normality test. Different sets of statistical tests were used for normally and non-normally distributed data. Pairwise statistical tests were performed using unpaired *t*-test with Welch’s correction for normally distributed data; they were performed using Mann-Whitney U test for non-normally distributed data. Where two-way analysis of variance (ANOVA) was used, a Bonferroni correction was used for *post hoc* pairwise comparisons. Kolmogorov-Smirnov tests were applied for cumulative frequency distributions. In all cases, means (or medians) were considered significantly different if the test reported *p* < 0.05. Most statistical tests and all graphing were done using Prism (GraphPad Software). Two-way ANOVA was done in MATLAB (The MathWorks).

## Results

**Figure 1:**
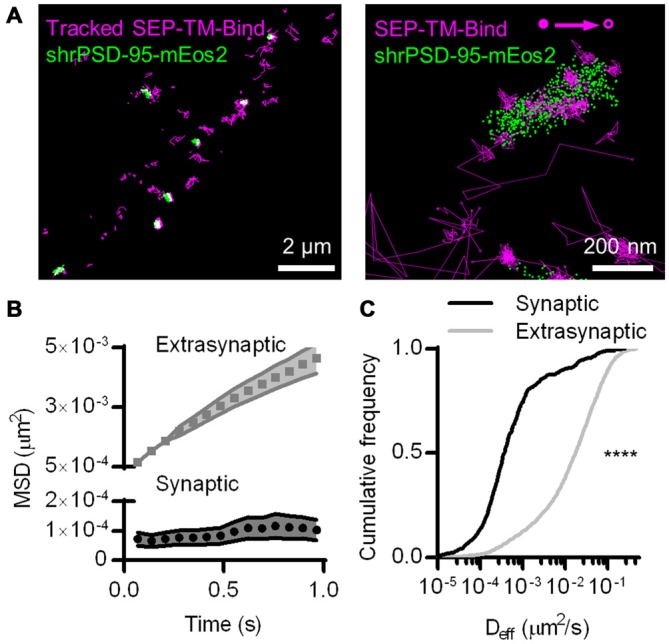
**Photoactivated localization microscopy (PALM)-point accumulation-for-imaging-in-nanoscale-topography (PAINT), single-molecule tracking during PALM imaging. (A)** (Left) Super ecliptic phluorin (SEP)-TM-Bind molecules tracked for at least eight frames super-imposed on molecules of shrPSD-95, accumulated from 5000–20,000 frames. (Right) A typical example of tracked probes superimposed on positions of shrPSD-95 molecules. The first and last localized positions are indicated as filled and open circles, respectively. **(B)** Mean squared displacement (MSD) over time of connected sub-segments of tracks (subtracks) that are lasted at least 15 frames (*n* = 156 synaptic and 2907 extrasynaptic tracks/113 PSDs/13 fields/11 cells/3 cultures). **(C)** Cumulative frequency distributions of D_eff_ for subtracks that are at least eight frames long (*n* = 654 synaptic and 3349 extrasynaptic tracks/113/13/11/3). ****p < 0.0001.

To perform single-molecule tracking during superresolution imaging of the PSD, we co-transfected 13–17 DIV hippocampal neurons with two cDNA constructs. The first encoded a single-pass TM protein composed of an extracellular SEP, the TM domain, and the intracellular carboxy terminus of stargazin, which enables this protein to bind PSD-95. Thus, we refer to it as SEP-TM-Bind (Li et al., 2016). This was co-transfected with shrPSD-95-mEos2, which expresses shRNA targeting PSD-95 along with an RNAi-resistant, mEos2-tagged PSD-95 (MacGillavry et al., 2013). To track motion of SEP-TM-Bind at the cell surface, we used uPAINT and applied anti-GFP nanobodies carrying Atto647N in the chamber to a final concentration of 0.5–2 nM. Concurrently, we localized the positions of PSD-95-mEos2 using conventional sptPALM methods (Figure 1A).

The SEP-TM-Bind probe exhibited clearly different mobility inside and outside of the PSD (Figure 1A right), as expected based on the behavior of AMPA-type glutamate receptors (Bats et al., 2007; Hoze et al., 2012), intercellular adhesion molecules such as neuroligin and leucine rich repeat transmembrane neuronal 2 (LRRTM2; Chamma et al., 2016), and NMDA-type glutamate receptors (Dupuis et al., 2014). We compared the diffusion patterns of molecules inside and outside of the PSD, when the PSD border was defined by the convex-hull border of PSD-95 positions. Probes outside of the PSD displayed near-free diffusion as evidenced by an almost linear plot MSD (Figure 1B). However, probes within the PSD showed a much slower mobility and appeared highly confined in their motion, as evidenced by saturation of the relationship between MSD and time. This curve approached a plateau of 104 ± 35 nm^2^, suggesting confinement within <60 nm diameter regions of the PSD (Ehlers et al., 2007). The effective diffusion coefficients (D_eff_) of the synaptic subtracks were ~2 orders of magnitude slower than those of the extrasynaptic subtracks (Figure 1C). This differential is similar to that seen for AMPARs (Bats et al., 2007; Hoze et al., 2012), suggesting that AMPAR motion within synapses likely is regulated by mechanisms that also impact many other types of molecules.

**Figure 2:**
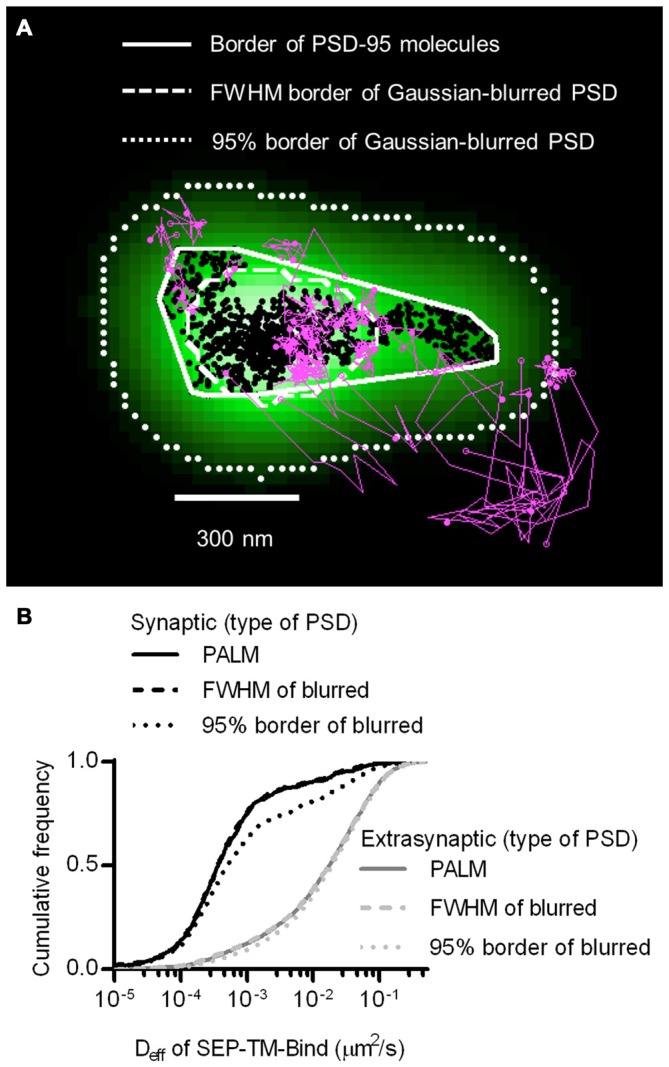
**Better discrimination of postsynaptic density (PSD) border reveals strong reduction of mobility within synapses. (A)** Typical tracks of the binding probe (magenta) and positions of shrPSD-95 (black circles) super-imposed. Convex hull border of PSD-95 positions (solid line), full-width half max (FWHM) border of Gaussian-blurred shrPSD-95 positions (dashed line), and full-width 5% max (95%) border (dotted lines). **(B)** Cumulative frequency distribution of synaptic (black lines) and extrasynaptic (gray lines) tracks segregated using the synaptic borders of PSDs determined using PALM’ed shrPSD-95 (solid lines), FWHM of Gaussian-blurred shrPSD-95 positions (dashed lines), or full-width 5% max (95% border) of the Gaussian-blurred shrPSD-95 positions (dotted lines) *(n* = 654 synaptic/3349 extrasynaptic tracks for PALM’ed PSDs, 658/3345 FWHM, 1197/3125 95% border; 113 PSDs, 11 cells, 3 cultures).

The improved resolution of the PSD border obtained by imaging the positions of individual PSD-95 molecules as opposed to using widefield or confocal microscopy should improve discrimination of which molecules are within the synapse. The average PSD area (0.085 ± 0.006 μm^2^, *n* = 263 PSDs/21 neurons) was within the ranges as previously detected by PALM (MacGillavry et al., 2013; Nair et al., 2013) and by electron microscopy (Harris and Stevens, 1989; Schikorski and Stevens, 1997; Shinohara et al., 2008). To test whether diffraction-limited PSD borders could perform as well as PALM of PSDs at segregating synaptic and extrasynaptic probes, we simulated diffraction by blurring the PSD-95 molecular density maps with a Gaussian point-spread function. Taking the FWHM of this intensity distribution as the border of the diffraction-blurred PSDs, as was done by Li et al. (2016), performed nearly as well as the PALM’ed PSD border in segregating synaptic and extrasynaptic probe movements. Taking the 95% border of the blurred PSDs diminished the difference between synaptic and extrasynaptic D_eff_ (Figure 2). Thus, how the PSD border is defined in diffraction-limited approaches can influence the accuracy of segregating diffusing molecules in different sub-compartments of the cell.

### Synaptic TM Protein Diffusion is not Influenced by PSD Size or Whole-Synapse PSD-95 Density

**Figure 3:**
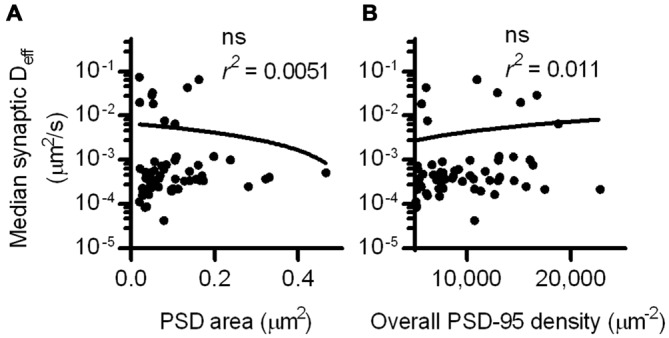
**PSD size and density do not correlate with intrasynaptic mobility. (A)** PSD area and median D_eff_ within each of the PSDs; linear regression test (*n* = 113 PSDs/11 cells/3 cultures). **(B)** PSD-95 density and median D_eff_ within each of the PSDs; linear regression test (*n* = same as in **A**).

Interestingly, the D_eff_ distribution within different synapses varied widely, and individual molecules exhibited D_eff_ spanning more than five orders of magnitude. This difference did not stem from neuron-to-neuron variability, as the median synaptic D_eff_ of the binding probes measured in different neurons differed by only up to 2-fold. We reasoned that this broad range of D_eff_ may arise because the diffusion environment within the PSD might vary based on synaptic size or geometry, or because the density of binding sites could influence how likely a probe is able to be bound at any given time. To test this, we first examined the relationship between the area of the PSD and the median D_eff_ of probes found within it. Based on linear regression analysis, we found no statistically significant correlation (Figure 3A). However, our previous study found that the fluorescence recovery of these probes after photobleaching spines was negatively correlated with PSD area (Li et al., 2016). Combined with this finding, this suggests that the size of the synapse correlates with the rate with which these probes enter and exit the spine, but does not influence their diffusion within the synapse. To determine whether overall PSD-95 density within the synapse can determine the diffusion of the binding probes, we examined the relationship between the density of PSD-95-mEos2 localizations and the synaptic median D_eff_ of probes in each PSD. The absolute density of localizations in all calculations was adjusted by the average expected number of blinks (one) of mEos2 in our experimental conditions (Annibale et al., 2011; MacGillavry et al., 2013). It should be noted that this measure of density does not incorporate the unknown fraction of total PSD-95 molecules that were mapped, and also ignores the numerous other binding partners of SEP-TM-Bind that may not correlate with the measured density of PSD-95-mEos2 as well as the likelihood of slight overexpression compared to endogenous protein level (~1.4×, see MacGillavry et al., 2013). Nevertheless, we found no statistically significant correlation (Figure 3B), suggesting that, given these caveats, the overall density of PSD-95 within the PSD does not influence the median diffusion of binding TM proteins in the synapse.

### The Control of TM Protein Diffusion by Binding and Steric Hindrance Within the PSD

Though the overall measured density of PSD-95 did not correlate with the diffusion coefficient of SEP-TM-Bind, it would be surprising if this key scaffolding protein did not affect the mobility of its binding partners at all. We thus considered that the distribution of PSD-95 molecules is highly heterogeneous within single synapses (Fukata et al., 2013; MacGillavry et al., 2013; Nair et al., 2013; Broadhead et al., 2016) and can display multiple regions of high density within the synapse. Namely, two synapses of the same PSD-95 density can have very different arrangement of PSD-95 molecules, an organization that could obscure the effect that PSD-95 molecular density can have on the diffusion of probes when measured at the level of the entire synapse. Consistent with this notion, computer modeling has demonstrated that measured arrangements of PSD-95 molecules can prevent a larger fraction of TM proteins from escaping the synapse than homogeneously distributed PSD-95 molecules, without changing the overall density of PSD-95 (Li et al., 2016).

**Figure 4:**
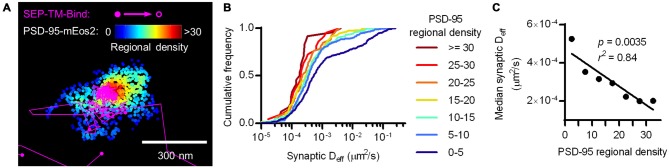
**Nanoscale regional density of PSD-95 within the synapse correlates with probe diffusion coefficient. (A)** Typical example of tracks and PSD-95 positions. Tracks pseudo-colored purple, PSD-95 positions pseudo-colored by regional density. **(B)** Cumulative frequency distributions of probe D_eff_ binned in increasing regional densites of PSD-95 surrounding the subtracks (*n* = 448 subtracks in 0–5 PSD-95, 281 in 5–10, 159 in 10–15, 106 in 15–20, 59 in 20–25, 22 in 25–30, 24 in 30+). **(C)** PSD-95 regional density and the median D_eff_ of the binding probe; relationship tested by linear regression.

To test whether the density of PSD-95 immediately surrounding the probe can influence its diffusion within the synapse, we defined a subsynaptic metric termed “regional PSD-95 density” to be the number of PSD-95 molecules surrounding a tracked probe position. We measured the regional density using a fixed radius based on the average positional error of the tracked molecules (30 nm, see “Materials and Methods” Section). However, the results of the following analyses depended only very weakly on the radius over the range of 15–80 nm (data not shown). We subdivided tracks into subsegments (subtracks) based on the regional PSD-95 density at each of their positions, and plotted the D_eff_ for subtracks based on their regional density. This analysis revealed that the higher the regional PSD-95 density, the slower the diffusion coefficients of the subtracks in that area of the PSD (Figures 4A,B). In fact, the median probe D_eff_ within the synapse was strongly correlated with the regional density of PSD-95 (Figure 4C).

### The Control of TM Protein Diffusion by Steric Hindrance Alone Within the PSD

**Figure 5:**
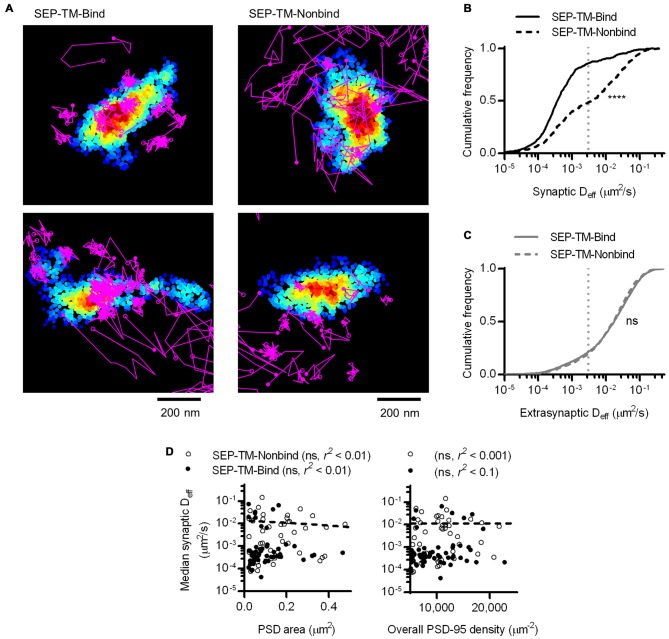
**A non-binding transmembrane protein enters and slows within the synapse, but not as much as if it can bind PSD-95. (A)** Typical examples of tracked probes (purple) superimposed on shrPSD-95 positions (pseudo-colored on a scale of regional density same as Figure 4A; binding probe SEP-TM-Bind (left), nonbinding probe SEP-TM-Nonbind (right). **(B)** Cumulative frequency distributions of the binding probe and nonbinding probe D_eff_ within PSDs (*n* = 654 tracks/113 PSDs/11 cells/3 cultures for SEP-TM-Bind, 519/91/10/3 SEP-TM-Nonbind). **(C)** Cumulative frequency distributions of the binding probe and nonbinding probe D_eff_ outside of PSDs (*n* = 3349 tracks for SEP-TM-Bind, 4470 SEP-TM-Nonbind). **(D)** (Left) PSD area and median D_eff_ within each of the PSDs; linear regression test (*n* = 91 PSDs/10 cells/3 cultures of SEP-TM-Nonbind, same as in Figure 3 for SEP-TM-Bind). (Right) Overall synaptic PSD-95 density and median D_eff_ within each of the PSDs; linear regression test (*n* = as in left panel).

At a first glance, this result may not be surprising, as it supports the idea that the more scaffold binding partners there are in the synapse, the more likely the probe will be bound and thus immobilized before diffusing further. However, this effect is more difficult to interpret if we consider that PSD-95 not only binds this probe, but can serve as a steric obstacle. In fact, PSD-95 is a hub for binding many other proteins that can serve as additional obstacles which could, without binding the probe, hinder its diffusion. To isolate the effect of steric hindrance from the combined effect of steric hindrance and probe-scaffold binding, we performed PALM-PAINT on a probe variant that cannot bind to PSD-95 (SEP-TM-Nonbind from Li et al., 2016). Interestingly, SEP-TM-Nonbind still entered synapses and diffused within them, but did not enrich within the PSD nearly as greatly as SEP-TM-Bind (Figure 5A and see also Li et al., 2016). Notably, the diffusion of this nonbinding probe within the synapse was dramatically faster than that of the binding probe (Figure 5B). On the other hand, the extrasynaptic diffusion of the nonbinding probe was not different from that of the binding probe (Figure 5C). It is interesting to note that using sptPALM (i.e., by photoactivating and tracking an mEos3 fusion protein rather than using an anti-GFP PAINT approach as here) there was a very similar mobility differential between the binding and nonbinding probes despite a substantial absolute difference in D_eff_ arising from the poorer localization precision of the fusion protein compared to the organic dye (Li et al., 2016). Thus, the mobility difference between the two probes is quite robust. Intriguingly, the shoulder-like shape of the cumulative D_eff_ distribution suggests that probes undergo multiple influences on their diffusion within the synapse. However, neither PSD area nor whole-synapse PSD-95 density correlated with probe diffusion within the PSD (Figure 5D).

By labeling SEP-tagged TM proteins with nanobodies, we add minimal but appreciable bulk to the extracellular domain of the diffusing entity. SEP is fused to the extracellular domain of the TM probes, adding approximately 3–5 nm of bulk; nanobody labeling of the GFP adds an additional 3–5 nm of bulk. While additional extracellular bulk has previously been shown to slow receptor diffusion (Groc et al., 2007), we expect the effect to be identical for both the binding and the nonbinding probes and thus not change our conclusions about the effect of postsynaptic steric hindrance.

**Figure 6:**
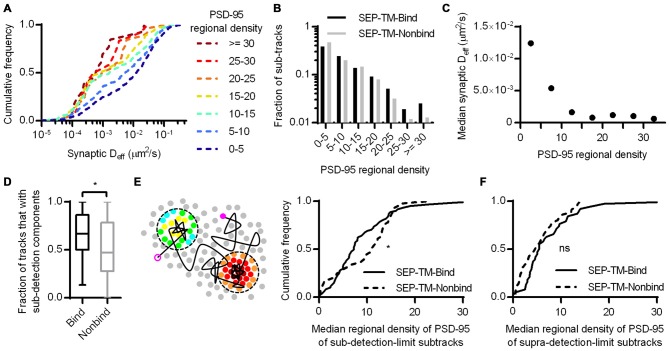
**Subsynaptic regional density of PSD-95 influences the mobility of a probe that does not bind PSD-95. (A)** Cumulative frequency distributions of the nonbinding probe D_eff_ binned in increasing regional densites of PSD-95 surrounding the subtracks (*n* = 480 subtracks in 0–5 PSD-95, 204 in 5–10, 148 in 10–15, 81 in 15–20, 32 in 20–25, 12 in 25–30, 13 in 30+). **(B)** Fraction of subtracks in different regional densities of PSD-95. **(C)** PSD-95 regional density and the median D_eff_ of the nonbinding probe. **(D)** Fraction of tracks with subsegments that were slowed below the detection limit per PSD (*n* = 113 PSDs for SEP-TM-Bind, 91 SEP-TM-Nonbind; *p = 0.0123 Mann-Whitney *U* test). **(E)** (Left) Cartoon highlighting the PSD-95 molecules surrounding subtrack durations that diffused below the detection limit. The open and closed purple circles indicate the beginning and end of a track, the circles pseudo-colored by regional density were within 30 nm of sub-detection limit subtracks. We calculated the median regional density of PSD-95 of all subtrack durations that diffused below the detection limit with every PSD. (Right) Median regional density of PSD-95 surrounding subtracks that were below the detection limit (*n* same as in **D**; *p = 0.0325 K-S test*)*. **(F)** Median regional density of PSD-95 surrounding subtracks that above the detection limit (*n* same as in **D**; ns, Not significant, *p* = 0.158 K-S test).

We next considered whether the regional density of PSD-95 immediately surrounding the nonbinding probe can sterically control the probe diffusion. To test this, we first subdivided the tracks into subtracks and binned them into increasing regional densities of PSD-95 molecules, as in Figure 4B. This revealed that despite the lack of a PSD-95-binding motif, the probe still diffused more slowly within higher density regions of the PSD (Figure 6A). The effect appeared to saturate at low D_eff_ since the mobility of these slowly moving molecules is below our detection limit (0.003 μm^2^/s).

If steric obstruction influences probe mobility, it may influence the overall pattern of probe position within the synapse. We thus compared the fraction of subtracks found in different regional PSD-95 densities (Figure 6B). Interestingly, though the binding and the nonbinding probes were distributed similarly through most density values, the nonbinding probes appeared to be preferentially excluded from the highly dense subregions (i.e., >25 regional molecules) of the synapse. Because of the small number of subtracks in these bins, however, this difference was not significant. Note also that the distribution of tracked molecules may not completely faithfully represent the total steady-state distribution of probe molecules, since molecules immobilized in the synapse for long periods are less likely to be recognized by a nanobody and be tracked by uPAINT.

It appeared that regional densities of PSD-95 higher than 10 had minimal effect on diffusion of the nonbinding probe in the D_eff_ range below our detection limit (Figure 6C), whereas the regional density of PSD-95 linearly correlated with the diffusion of the binding probe (Figures 4B,C). Thus we wondered whether the two probes required different degrees of steric hindrance in order to be stabilized. To test this, we first noted that during multiple visits to a synapse, or even within a single visit, probe molecules could display both slow and fast periods of motion. Considering only the subtracks within synapses, SEP-TM-Bind had a higher fraction than SEP-TM-Nonbind of these subtracks for which D_eff_ was below the detection limit (Figure 6D). This indicates that the binding probes were more often slowed down while within the synapse than were the non-binding probes. We could then further exploit the ability afforded by PALM with uPAINT to examine the very local environment of the molecules as they moved within the synapse. Specifically, we noted that if steric hindrance slows mobility of the both the binding and non-binding probes, but binding is only able to slow SEP-TM-Bind, then SEP-TM-Bind would be expected to show a greater tendency to slow its mobility in relatively less dense PSD subregions. That is, even sparse binding partners could potentially capture and immobilize SEP-TM-Bind whereas higher concentrations of molecules would be required to sterically obstruct SEP-TM-Nonbind. Consistent with this, when we analyzed the sub-detection-limit portion of the nonbinding probe subtracks, we found that these were preferentially in locales of higher regional density of PSD-95 compared to those of the binding probes (Figure 6E). Interestingly, the fraction of the subtracks above the detection limit did not show any difference in regional PSD-95 density (Figure 6F), with even a trend to the opposite relationship.

**Figure 7:**
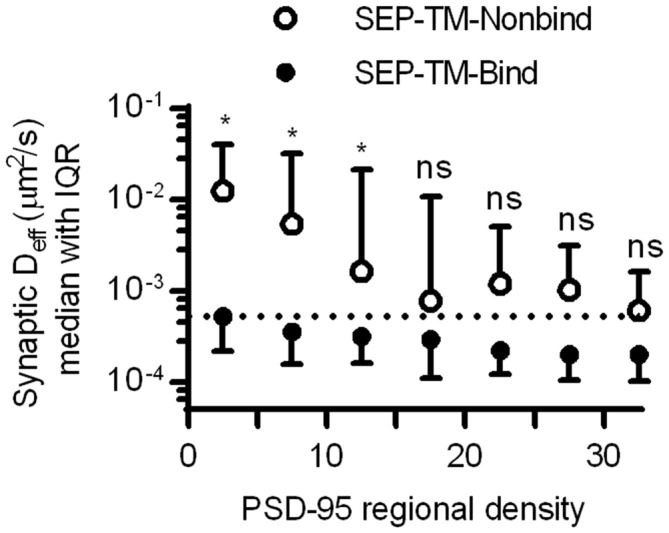
**Estimating how dense PSD-95 is when protein mobility is slowed sterically.** D_eff_ of SEP-TM-Nonbind and of SEP-TM-Bind within synaptic subregions of different PSD-95 densities (*n* of SEP-TM-Nonbind same as in Figure 6B; that of SEP-TM-Bind same as in Figure 4B; two-way ANOVA, effect of binding *F*_1,6_ = 15.27, *p* < 0.001; effect of regional PSD-95 density *F*_1,6_ = 13.53, *p* < 0.0001; *post hoc* Bonferroni multiple comparisons tests between probes in increasing regional densities *p < 0.05, ns, Not significant).

Altogether, these results suggest that crowding by scaffold molecules and perhaps other proteins is sufficient to stabilize TM proteins in the absence of binding. How dense does the molecular environment have to be in order to slow the TM probes sterically as much as the combined influence of steric hindrance and probe-scaffold binding? To estimate an answer to this question, we compared the diffusion coefficients of the binding and nonbinding probes within increasing regional densities of PSD-95 (Figure 7). As expected based on Figures 4, 6, the synaptic D_eff_ of both probes decreased gradually with increasing PSD-95 regional density. However, the D_eff_ of SEP-TM-Nonbind decreased precipitously over the range of 0–15; yet, it did not decrease further at higher densities. Furthermore, the D_eff_ of SEP-TM-Nonbind plateaued at the D_eff_ value displayed by SEP-TM-Bind at very low PSD-95 densities. Thus, by this analysis, ~15 PSD-95 molecules per region of 30 nm radius (~5000 molecules/μm^2^) is the threshold beyond which the steric hindrance is as strong as both steric and binding influences combined.

## Discussion

Using simultaneous single-molecule tracking and localization microscopy enabled by uPAINT and PALM, we demonstrated that the subsynaptic regional density of a scaffold protein PSD-95 can stabilize the surface membrane diffusion and positional organization of a single-pass transmembrane protein probe. The denser the regional density of PSD-95, the slower was the diffusion of the TM probe. This influence was apparent even in the absence of probe-scaffold binding, indicating steric hindrance by macromolecular crowding can complement protein-protein binding interactions in organizing TM proteins within the synapse.

### The Roles of Receptor-Scaffold Binding and Macromolecular Crowding in Subsynaptic Organization

The mobility of AMPARs in the synapse is increased when the binding of their accessory subunit Stargazin to PSD-95 is disrupted (Bats et al., 2007; Sainlos et al., 2011), providing strong evidence that receptors are acutely stabilized by PSD-95 binding. However, even in these conditions some stabilization of receptors in the synapse occurs, and our results indicate specifically that even small probes carrying a cytosolic tail unable to bind PSD-95 is still slowed substantially in the synapse. What controls this stabilized fraction even in the absence of binding has been a mystery. Mechanisms such as additional binding interactions have been proposed, which is not unlikely considering that AMPARs have numerous auxiliary subunits (Tomita et al., 2003; Cho et al., 2007; Soto et al., 2009; Kalashnikova et al., 2010; von Engelhardt et al., 2010; Erlenhardt et al., 2016) that can bind to various scaffolding proteins. However, even a cytosolic domain composed of just a GFP-type molecule is slowed within the synapse (Li et al., 2016). Thus, we propose a more general mechanism that likely applies not only to glutamate receptors but also to other TM proteins critically important for synaptic function. In this model, receptor-scaffold binding is a ticket to entry and exit; PSD morphing redistributes even bound receptors within the synapse; and macromolecular crowding in combination with binding stabilizes the receptors at subsynaptic domains highly packed with other proteins important for synaptic transmission.

Interestingly, though the TM binding probe is much smaller than AMPARs, its distribution of D_eff_ within the synapse appeared as slow as, or even slower than, AMPAR diffusion measured in other studies using dye-conjugated antibodies or quantum-dot-conjugated nanobodies (Nair et al., 2013; Cai et al., 2014). It is possible that these various labeling approaches preferentially sample receptors that exit the synapse and diffuse more freely in the perisynaptic space, which may tend to obscure real differences in the relative numbers of low-D_eff_ molecules that occupy the synapse for long periods. On the other hand, taken at face value, the similarity is consistent with our previous report using sptPALM that a substantial and nearly identical fraction of mEos-tagged AMPARs and binding probes diffused with D_eff_ < 0.02 μm^2^/s (Li et al., 2016), further supporting the notion that much of the synapse is so crowded it stabilizes and organizes both large and small TM proteins.

Previously, we demonstrated using partial synapse Fluorescence Recovery after Photobleaching (FRAP), smtPALM alone, and uPAINT alone that the protein bulk of the TM probe decreased its diffusion within the synapse. However, previous approaches were limited in ability to assess how the complex structure within the PSD could influence the mobility and organization of TM proteins in a living synapse. Results from PALM-PAINT indicate that a particular degree of postsynaptic crowding, which we estimate as 5000 molecules/μm^2^, can be sufficient to stabilize TM protein diffusion in the absence of binding. This density translates to an average ~14 nm inter-PSD-95 distance, very similar to the mean nearest-neighbor distance of ~13 nm between the “vertical filaments” corresponding to PSD-95 as measured in EM tomography (Chen et al., 2008). Note that we deduce this as an average spacing, but could not directly measure it around individual moving probes. The similarity between these values suggests that rather subtle variations in scaffold density across the lateral extent of the synapse could change TM protein mobility substantially. This high density packing is similar to what has been measured for AMPA receptors (e.g., 2000–4000/μm^2^, see Levet et al., 2015). Indeed, receptor-scaffold binding may facilitate the assembly of this tight packing. Though the fractional time synaptic AMPARs spend bound to PSD-95 is not known, macromolecular crowding is likely to augment maintenance of this architecture once assembled, because receptors in crowded areas that dissociate from scaffolds will face a longer escape time from the region and thus are more likely to rebind PSD-95.

If domains of high PSD-95 density tend to accumulate not only probes such as used here but also receptors, then their impact on synaptic transmission will depend on where they are with respect to sites of neurotransmitter release (MacGillavry et al., 2011). Recently, we have found that nanoclusters of PSD-95 frequently align transsynaptically with sites of neurotransmitter release, as indicated by their transsynaptic alignment with RIM1/2 molecules which in turn correlate with presynaptic vesicle fusion locations (Tang et al., in press). Thus, we speculate that the enhanced crowding within these high-density subdomains will slow and help limit the escape of receptors from points in the synapse where they are most likely to be activated during neurotransmission.

Crowding within high-density subdomains is likely not due only to postsynaptic scaffolding proteins. Indeed, transmembrane adhesion molecules, which associate with one another across the synaptic cleft, will enhance crowding further in the extracellular, transmembrane, and intracellular domains. The distribution of these adhesion molecules may regulate the alignment between neurotransmitter receptors and release sites, though this is not known. The TM probes employed here share commonalities with some of the intercellular adhesion molecules in size (e.g., single-pass TM proteins) or the ability to bind PSD-95, yet synaptic adhesion proteins display quite divergent patterns of expression within the synapse. SynCAM is distributed in clusters surrounding the border of PSD-95 molecules (Perez de Arce et al., 2015), as are some members of the cadherin/catenin system (Uchida et al., 1996). On the other hand, neuroligin1 and LRRTM2 are inside the synapse and distributed in clusters reminiscent of PSD-95 (Chamma et al., 2016).

Surprisingly, LRRTM2, though smaller in both extracellular and intracellular length than neuroligin1, in fact diffuses slower than neuroligin1 (Chamma et al., 2016). Moreover, LRRTM2 is more compactly distributed and in the synaptic center than the larger neuroligin1, suggesting that LRRTM2 is more likely associated with dense regions of PSD-95 which are often found near the center of the PSD (MacGillavry et al., 2013; Tang et al., in press). This paradoxical result confirms that the arrangements of TM proteins cannot be predicted by their bulk alone. Interestingly, however, AMPARs associate with LRRTM2 through their extracellular domains (de Wit et al., 2009), potentially regulating the mobility of each protein by the addition of further bulk and protein-protein interactions. Further experiments are needed to tease out how the various interactions on diverse synaptic TM protein species dictate one another’s spatial arrangement.

The diffusion of TM proteins appeared complicated outside of the synapse particularly within few hundred nanometers from the border of the PSD. In some cases, the nonbinding probe moves over a large area in the extrasynaptic regions, but in other cases, they can appear confined or immobilized as the binding probes. Proteins other than scaffolding proteins can certainly affect the mobility of these probes outside the synapse. Some factors could affect both probes roughly equally: regions with high density of endocytic adaptor molecules (Blanpied et al., 2002; Petralia et al., 2003; Racz et al., 2004), zones of dense cortical cytoskeleton (He et al., 2016), sites of plasma membrane-ER apposition (Spacek and Harris, 1997). In addition, puncta adherens or clusters of adhesion molecules (Perez de Arce et al., 2015) may slow transit of all TM proteins. Less frequently, undetected regions could exist that would selectively affect the binding probe. For example, small and low-density regions below the PSD detection criteria (<10 molecules and <30 nm in diameter) could be situated within nanometers of the detected PSD border. These could come from small segments of multi-segmented PSDs (Spacek and Hartmann, 1983; Stewart et al., 2005), but these types of PSDs are rare. In addition, though the density of PSD-95 outside the PSD border is quite low (Zhang and Diamond, 2009; Perez de Arce et al., 2015), its extrasynaptic mobility has not been measured and there may be enough PSD-95 in the perisynaptic region to bind and immobilize the binding probes. We speculate that the overall extrasynaptic diffusion of both probes are not different because few extrasynaptic regions selectively affect the binding probe but not the nonbinding probe.

### Role of Crowding in Synaptic Plasticity

The many types and time scales of ongoing and triggered PSD plasticity that have been documented (Okabe et al., 1999; Blanpied et al., 2008; MacGillavry et al., 2013; Bosch et al., 2014) suggest that PSD reorganization during plasticity will affect accumulation not just of TM proteins like AMPARs but additional molecules contributing to crowding as well. For instance, PSD-95 content in spines has been shown to decrease transiently after an LTP induction protocol in hippocampal slice cultures (Steiner et al., 2008). This may directly lead to loss of AMPARs as their binding partners are lost. However, the loss of PSD-95 may decrease crowding, which could prompt a net loss of even TM proteins with minimal direct binding to PSD-95 (e.g., desensitized AMPARs uncoupled from stargazin Constals et al., 2015). This further loss of molecular crowders could then further facilitate receptor exit. However, whether the transient loss of spine PSD-95 reflects a disruption of high-density areas of the synapse is unknown.

On the other hand, speculating further, if the transient decrease in synaptic PSD-95 during LTP induction reflects primarily loss from the PSD edge, and thus is not correlated with significant de-crowding at high-density regions, then the continued presence of additional nonbinding proteins at these high-density regions could in fact obstruct the entry of AMPARs to these regions and limit the changes in AMPARs level, at least over certain kinetic phases. Thus, it would be tempting to speculate in this case that the nonbinding proteins could affect LTP induction kinetics but not maintenance, as morphing dynamics and internal mixing of the PSD would eventually enable synapses to reach their new steady state capacity of AMPARs on a time scale of minutes. Resolving these many possibilities in the future will require close examination of the kinetics of protein redistribution and exchange during plasticity.

### Advantages and Disadvantages of PALM-PAINT

PALM of the PSD border improves discrimination of those molecules definitively within the synapse proper. However, we suspect that the effect of crowding may have been underestimated in our analysis because spatial and temporal alignment of the uPAINT and PALM data was subject to residual errors that may have diminished a larger underlying effect. The two color channels faced an alignment error of ~6 nm, which would somewhat blur our measurement of regional PSD-95 density around individual tracked locations. In addition, the uPAINT data is subject to error stemming from the finite precision of individual localizations. The Atto647N we used for tracking is a relatively bright organic dye and helps to maximize this precision and thus minimize error in the estimate of D_eff_. However, brighter, longer-lasting fluorophores could be advantageous. Nanobody-labeled small quantum dots (Wang et al., 2014) have been used to track AMPARs in and around synapses, and have the additional advantage of being so bright as to facilitate tracking in 3D (Cai et al., 2014). However, 3D mapping of the PSD would require high localization numbers and longer imaging durations (Legant et al., 2016; and see below), and the *z* resolution normally obtainable without 4pi detection is usually worse than 100 nm for fluorescent proteins, making this difficult to implement.

In our application of PALM-PAINT, there was only limited temporal relationship between individual tracks (generally lasting <1 s and the PALM map (aggregated over the imaging session of generally 4–6 min)). Though lateral drift was corrected during this time (to an error we estimated as <10 nm), ongoing morphing and internal reorganization of the PSD (Kerr and Blanpied, 2012; MacGillavry et al., 2013) presumably degraded many details of the PSD-95 distribution in our final images. The reduced precision in capturing the true regional density of PSD-95 molecules would diminish the difference we saw between probes in different regional densities, and also reduce the difference between binding and nonbinding probe. Further, probes in a similar subsynaptic space but tracked early vs. late in the mapping might have not truly experienced the same degree of steric hindrance. However, the differences we observed were robust even in the face of these errors. Ideally, to capture the true effect size, one would need to monitor lateral drift continuously (Bon et al., 2015) and achieve more rapid mapping (Huang et al., 2013). However, in structures with low protein copy number, a large fraction of the proteins must be mapped to achieve statistical reliability (MacGillavry et al., 2013; Legant et al., 2016), which may preclude time-lapse imaging except if the protein exchange rate is high compared to the photobleaching rate induced by imaging.

We hope the combined approach of PALM-PAINT will help answer many key questions regarding synapse architecture and plasticity. One key issue is what mechanisms assemble the particular organization of PSD-95, a pattern that appears to dictate receptor number and position (Opazo et al., 2012). One possibility is that the more deeply positioned multi-domain proteins in the PSD, such as the Shank and GKAP families (Valtschanoff and Weinberg, 2001; Dani et al., 2010), may establish a platform of loose spacing with which the more superficial proteins such as PSD-95 may interact (Chen et al., 2008). Interestingly, in this case, a close interaction of the deeper PSD with cytoskeleton (Frost et al., 2010; MacGillavry et al., 2016) may thus provide a link between activity-dependent plasticity of spine and PSD structure. Alternatively, cleft-resident adhesion molecules have distinct organizational patterns (Perez de Arce et al., 2015; Chamma et al., 2016), that may guide intracellular protein organization in both the presynaptic and postsynaptic cells. Dissecting these possibilities, which require nanoscale resolution of position and mobility of multiple proteins, may be aided by future PALM-PAINT applications.

## Author Contributions

TPL and TAB designed research; TPL performed research and data analysis; TPL and TAB wrote the article.

## Funding

This work was supported by the National Institutes of Health Grant F30MH102891 to TPL and R01MH080046 and R01MH096376 to TAB and the Kahlert Foundation to TAB.

## Conflict of Interest Statement

The authors declare that the research was conducted in the absence of any commercial or financial relationships that could be construed as a potential conflict of interest.
